# Identification of novel biomarkers involved in pulmonary arterial hypertension based on multiple-microarray analysis

**DOI:** 10.1042/BSR20202346

**Published:** 2020-09-16

**Authors:** Yi Ma, Shu-Shu Chen, Yan-Yan Feng, Huan-Liang Wang

**Affiliations:** 1 Department of Anesthesiology, Qilu Hospital of Shandong University, Jinan 250012, China; 2 Shenzhen Research Institute of Shandong University, Shenzhen 518058, China

**Keywords:** Bioinformatics, Computational biology, Pulmonary Arterial Hypertension, Transcriptomics

## Abstract

Pulmonary arterial hypertension (PAH) is a life-threatening chronic cardiopulmonary disorder. However, studies providing PAH-related gene expression profiles are scarce. To identify hub genes involved in PAH, we investigate two microarray data sets from gene expression omnibus (GEO). A total of 150 differentially expressed genes (DEGs) were identified by limma package. Enriched Gene Ontology (GO) annotations and Kyoto Encyclopedia of Genes and Genomes (KEGG) pathways of DEGs mostly included mitotic nuclear division, ATPase activity, and Herpes simplex virus one infection. Ten hub genes from three significant modules were ascertained by Cytoscape (CytoHubba). Gene set enrichment analysis (GSEA) plots showed that transcription elongation factor complex was the most significantly enriched gene set positively correlated with the PAH group. At the same time, solute proton symporter activity was the most significantly enriched gene set positively correlated with the control group. Correlation analysis between hub genes suggested that SMC4, TOP2A, SMC2, KIF11, KIF23, ANLN, ARHGAP11A, SMC3, SMC6 and RAD50 may involve in the pathogenesis of PAH. Then, the miRNA-target genes regulation network was performed to unveil the underlying complex association among them. Finally, RNA extracted from monocrotaline (MCT)-induced Rat-PAH model lung artery tissues were to conduct quantitative real-time PCR (qRT-PCR) to validate these hub genes. In conclusion, our study offers new evidence for the underlying molecular mechanisms of PAH as well as attractive targets for diagnosis and treatment of PAH.

## Introduction

Pulmonary arterial hypertension (PAH) is a common disorder worldwide characterized by irreversible remodeling of the distal pulmonary arteries, resulting in sustained rise pulmonary vascular resistance and right ventricular failure, eventually, death [1,2]. Over the past decades, tremendous progress has been made in understanding the basic pathobiological of PAH and its essential history, prognostic biomarkers, and treatment options. However, studies providing PAH-related gene expression profiles remain rare. In consequence, it is an urgent mission to identify clinical molecular biomarkers and investigate the underlying mechanisms involved in the PAH, that might help in developing novel scientific-based diagnostic and adopt target-treatment methods in PAH patients.

In current years, bioinformatics analysis has been widely used to analyze microarray data to determine differentially expressed genes (DEGs) and perform various analyses. However, on account of the small sample size and high false-positive rate in single microarray analysis, it may be hard access to reliable data-mining results. In our research, two messenger RNA (mRNA) microarray data sets acquired from gene expression omnibus (GEO) were prepared for further analyses. DEGs between pulmonary arterial hypertension patients and healthy controls were screened to identify vital biomarkers. Possible differentially expressed genes and hub genes involved in pulmonary arterial hypertension were investigated via Gene Ontology (GO) annotation, Kyoto Encyclopedia of Genes and Genomes (KEGG) pathway enrichment, protein–protein interaction (PPI) network analysis and Gene set enrichment analysis (GSEA). Eventually, a total of 150 DEGs and ten hub genes were screened, which may be prospective diagnostic biomarkers and target-treatment for PAH.

## Materials and methods

### Microarray data acquisition

**Figure 1:**
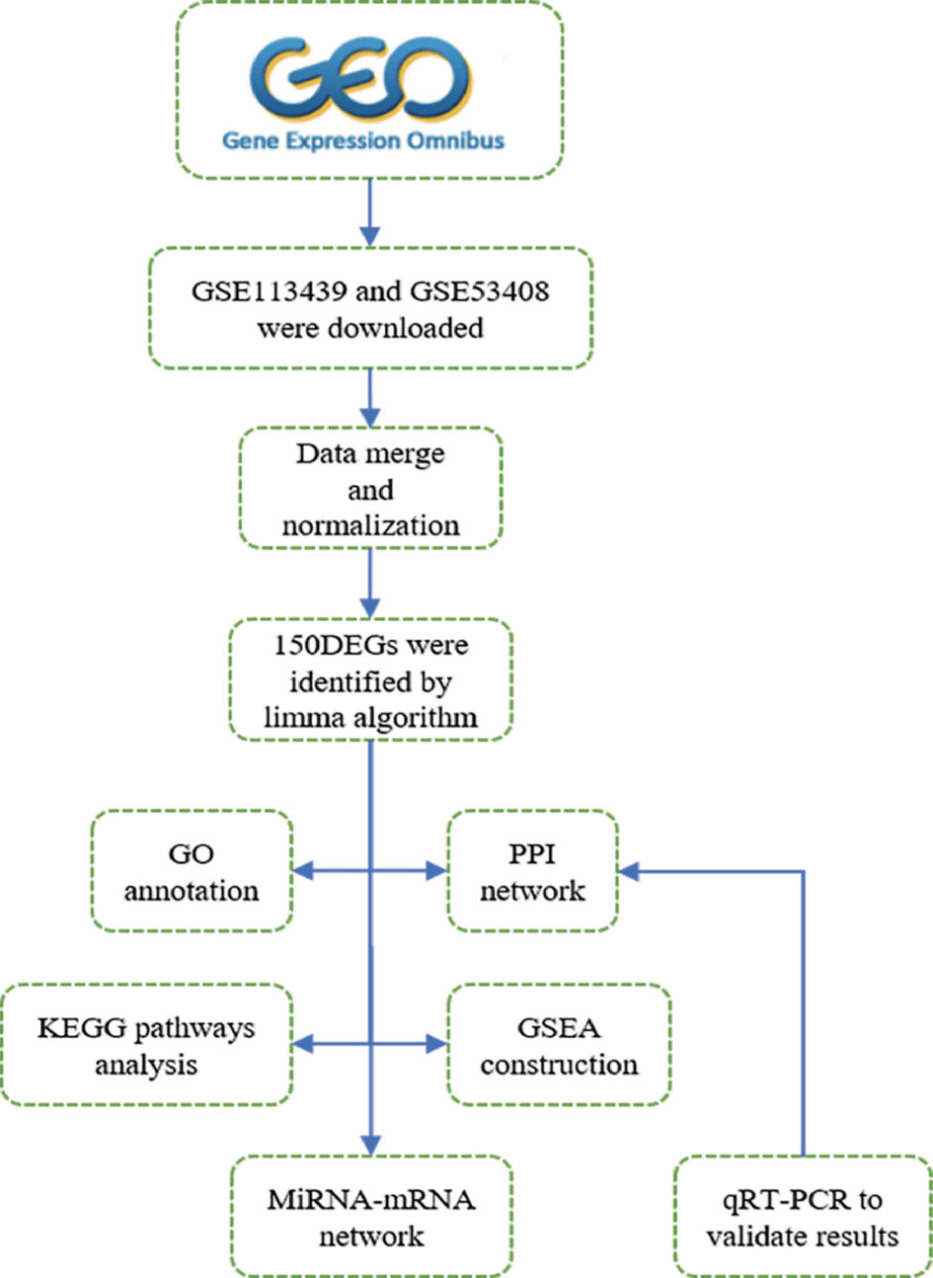
The workflow of our research GO, Gene Ontology; GSEA, Gene Set Enrichment Analysis; KEGG, Kyoto Encyclopedia of Genes and Genomes; PPI, protein–protein interaction.

Microarray data were downloaded from GEO (http://www.ncbi.nlm.nih.gov/geo), which is a public genomics database that contains sufficient high-throughput gene expression data [3]. Series matrix files and platform information of GSE113439 and GSE53408 were downloaded from the GEO database. GSE113439 is based on GPL6244 (Affymetrix Human Gene 1.0 ST Array [transcript (gene) version]) platform and includes 15 pulmonary arterial hypertension patients and 11 normal controls. GSE53408 is based on GPL6244 platform (Affymetrix Human Gene 1.0 ST Array [transcript (gene) version]) and collect 12 pulmonary arterial hypertension patients and 11 healthy controls. To make this article better understand, the data processing procedure of our research was illustrated in the workflow (see Figure 1).

### Data processing and DEGs determining

Data preprocessing included transform gene probes into gene symbols, data consolidation, and batch normalization. Probes without gene symbols or genes with more than one probe were deleted or averaged, respectively. The merged data were prepossessed by SVA package in R software (version 4.0.0) to remove batch effects [4]. limma package in R (version 4.0.0) software was utilized to screen DEGs between pulmonary arterial hypertension patients and normal controls, followed by performing data normalization [5]. Then, adjusted *P*<0.05 and |log FC| (fold change) > 1.5 were considered statistically significant. Heatmap package in R software (version 4.0.0) was employed to map the different profiles of DEGs.

### GO annotation and KEGG pathway enrichment analysis

The database for annotation, KEGG analysis, visualization and integrated discovery was performed via Clusterprofiler package and AnnotationHub package as well as AnnotationDbi package in R software (version 4.0.0) that provide systematic functional annotation information about genes and protein [6]. GO annotation, which includes biological processes (BP), cellular component (CC), and molecular function (MF) analyses [7]. KEGG is a popular online database used in pathway analysis [8]. Gene count >2 and *P*-value < 0.05 were recognized as the threshold. Both GO annotation and KEGG pathway analyses of DEGs were performed via R software (version 4.0.0).

### Gene set enrichment analysis

Gene set enrichment analysis (GSEA, Broad Institute, Inc., Massachusetts Institute of Technology, and Regents of the University of California) is a computational method used to assesses whether a predefined a set of genes shows statistically significant and consistent differences between two biological states [9]. In our study, GSEA software(version 4.0.3) was used to perform GO analysis on all detected genes and false discovery rate (FDR) <25% and *P*<0.05 were regarded as the cut-off criteria. The gene matrix in our analysis was c5.all.v7.1symbols.gmt[Gene ontology].

### PPI network construction and hub gene identification

Search tool for the retrieval of interacting genes (STRING 11.0; http://string-db.org) was applied to create a PPI network of DEGs [10]. Interaction with a combined score >0.7 was set as the cut-off point. Cytoscape (3.8.0) software was used to visualize the PPI network [11]. Significant modules and hub genes in the PPI network were identified by molecular complex detection (MCODE 1.6.1), a plug-in of Cytoscape (3.8.0) software that clusters a network based on the topology to recognize closely connected regions automatically [12]. The parameters of DEGs clustering and scoring were set as follows: MCODE score ≥ 4, degree cut-off = 2, node score cut-off = 0.2, max depth = 100, and *k*-score = 2.

### miRNA-gene network construction

The inter-regulated miRNA and ten hub genes were identified with the help of several open online tools, which including miRWalk, miRDB, and TargetScan. Cytoscape tool was applied to construct the interaction network between miRNA and target mRNA.

### Reverse transcription and quantitative real-time PCR (qRT-PCR)

Total RNA was isolated from MCT-induced Rats-PAH model (*n*=5) and control rats (*n*=5) lung artery tissues using TRIzol reagent (Invitrogen, U.S.A.), respectively. According to the manufacturer’s instructions, RNA quality and quantity were measured by spectrophotometer. Then RNA was reverse-transcribed into cDNA using PrimeScript™RT reagent kit (No. RR037A, TaKaRa, Japan). qRT-PCR was performed on a LightCycler 480 (Roche). The gene expression levels were analyzed using a TB Green Premix Ex Taq™ kit (No. RR420, TaKaRa, Japan). The relative expression of the gene was calculated using the 2^−ΔΔCt^ method. Tubulin was viewed as an internal control. The thermal cycler conditions were as follows: 30 s at 95.0°C for cDNA denatured, 40 cycles of 95.0°C for 5 s, 60°C for 30 s and 1 min at 60.0°C. Our experiment was conducted three biological replicates, and all primer sequences are listed in Table 4.

### ConnectivityMap (CMap) analysis

To explore potential drugs that may ameliorate pulmonary arterial hypertension, we processed DEGs via CMap analysis (https://portals.broadinstitute.org/cmap), which integrated diseases, drugs, genes based on gene expression profiles. In our study, mean < − 0.4 and *P*<0.01 were set as the screening criteria.

### Statistical method

Data are presented as mean ± standard. Graphs were drawn by using GraphPad Prism software (version 8.0, La Jolla, CA, U.S.A.). PAH-rat versus healthy rat data were analyzed by Student *t-*test with Welch correction. *P-*value < 0.05 were considered statistically significant.

## Results

### Detection of DEGs related to PAH

**Figure 2:**
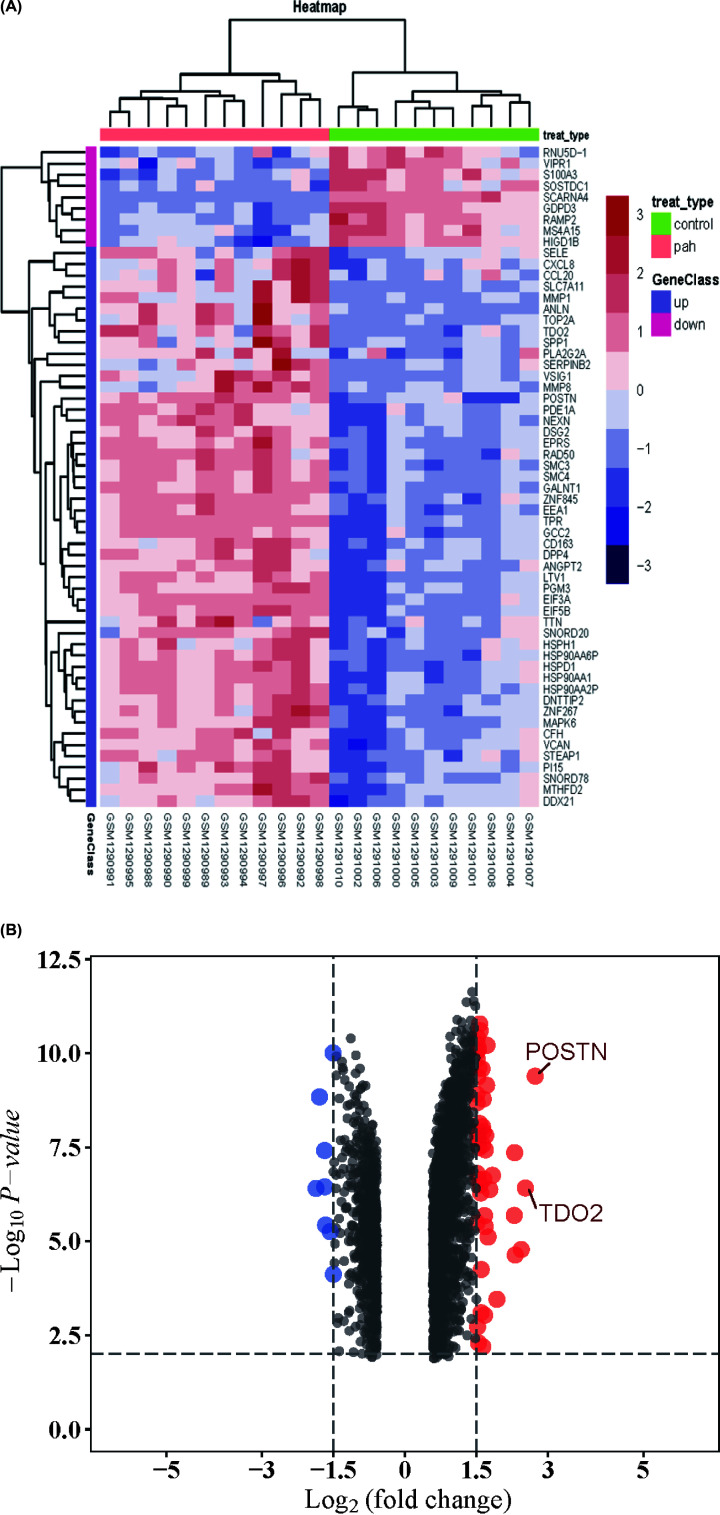
Heatmap and volcano plot of DEGs (**A**) Heatmap of 150 DEGs screened by limma package. Red areas represent highly expressed genes and green areas represent lowly expressed genes involved in PAH patients compared with healthy controls. (**B**) Volcano plot analysis and heatmap cluster of DEGs. Red dots represent up-regulated genes and green dots represent down-regulated genes in PAH patients compared with normal controls. Moreover, genes which log_2_FC>2 were marked; DEG, differentially expressed gene; PAH, pulmonary arterial hypertension.

To identify DEGs linked with PAH, we download two microarray expression profiles from GEO (GSE113439 and GSE53408). About 150 DEGs involved in PAH were screened by limma package after consolidation and normalization of the original microarray data (adjusted *P*-value < 0.05, |logFC| > 1.5). As was shown in the heatmap (Figure 2A), among them, eight genes were down-regulated, and 142 genes were up-regulated (Figure 2B).

### Gene set enrichment analysis

**Figure 3:**
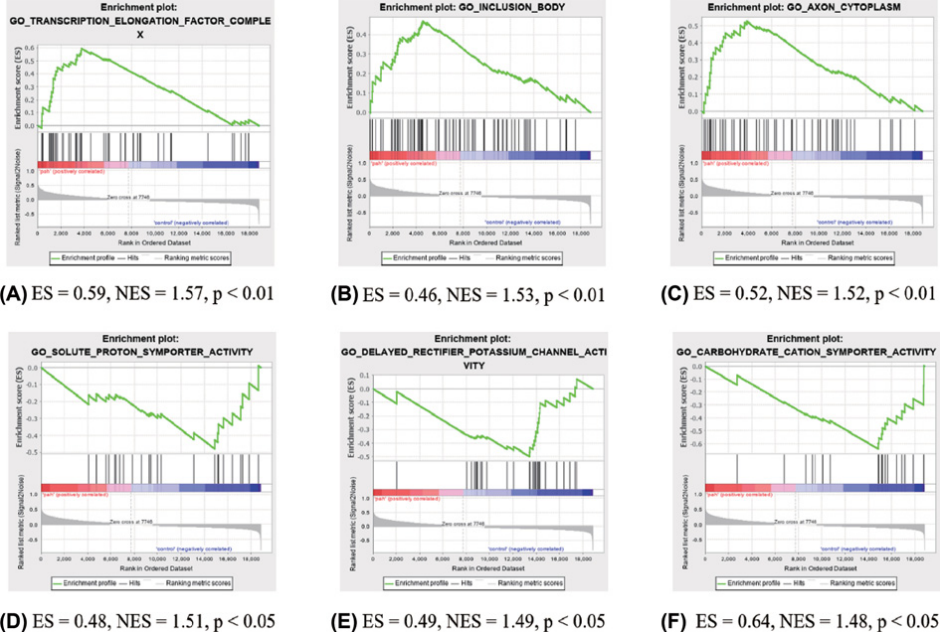
GSEA plot showing most enriched gene sets in the PAH group and healthy controls (**A**) The most significant enriched gene set positively correlated with the PAH group was transcription elongation factor complex. (**B**) The second meaningful enriched gene set positively correlated with the PAH group was inclusion body. (**C**) The third significant enriched gene set positively correlated with the PAH group was axon cytoplasm. (**D**) The most significant enriched gene set positively associated with healthy controls was solute proton symporter activity. (**E**) The second meaningful enriched gene set positively correlated with healthy controls was delayed rectifier potassium channel activity. (**F**) The third significant enriched gene set positively associated with healthy controls was carbohydrate cation symporter activity; ES, enrichment score; GSEA, gene set enrichment analysis; NES, normalized enrichment score; PAH, pulmonary arterial hypertension.

GSEA was performed to identify gene sets with a statistically significant difference between the PAH groups and healthy controls. Most significant enriched gene sets positively correlated with the PAH group included transcription elongation factor complex, inclusion body, and axon cytoplasm (Figure 3A–C). Most significant enriched gene sets positively correlated with the control group included solute proton symporter activity, delayed rectifier potassium channel activity, and carbohydrate cation symporter activity (Figure 3D–F).

### PPI network analysis and hub genes recognition

**Figure 4:**
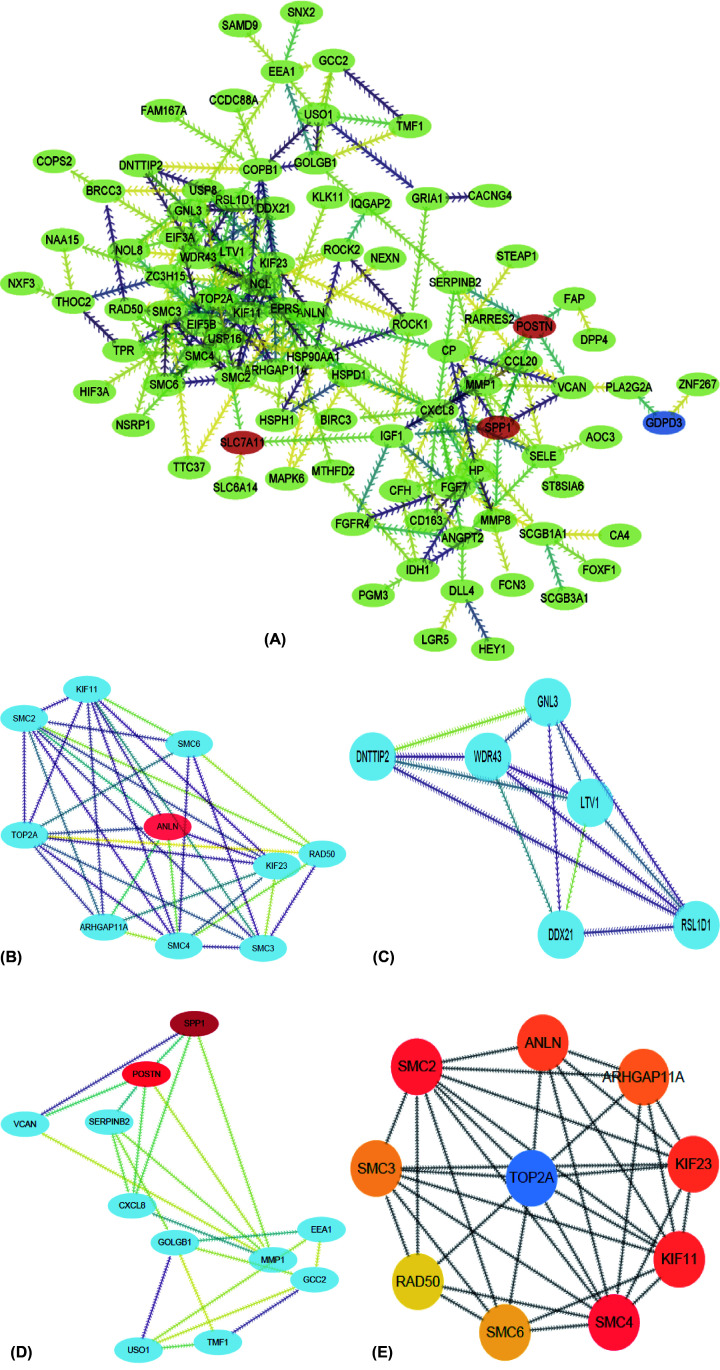
PPI network and three significant modules of DEGs (**A**) PPI network of DEGs created by STRING. Circles represent genes and lines represent PPIs. (**B**) The most significant module identified by MCODE (score = 6.857). (**C**) The second significant module identified by MCODE (score = 5.600). (**D**) The third significant module identified by MCODE (score = 4.400). (**E**) The top ten genes identified by cytoHubba; DEG, differentially expressed gene; PPI, protein–protein interaction.

To identify the most significant clusters of DEGs, the PPI network of DEGs was constructed by STRING and visualized by Cytoscape (3.8.0). As was shown in Figure 4A, 101 nodes and 230 edged were contained in the PPI network. Three most significant modules were recognized by MCODE, a plug-in of Cytoscape (Figure 4B–D). Then, ten hub genes closely linked to pulmonary arterial hypertension were identified by using cytoHubba, a plug-in of Cytoscape (Figure 4E and Table 3).

### GO enrichment analysis

**Figure 5:**
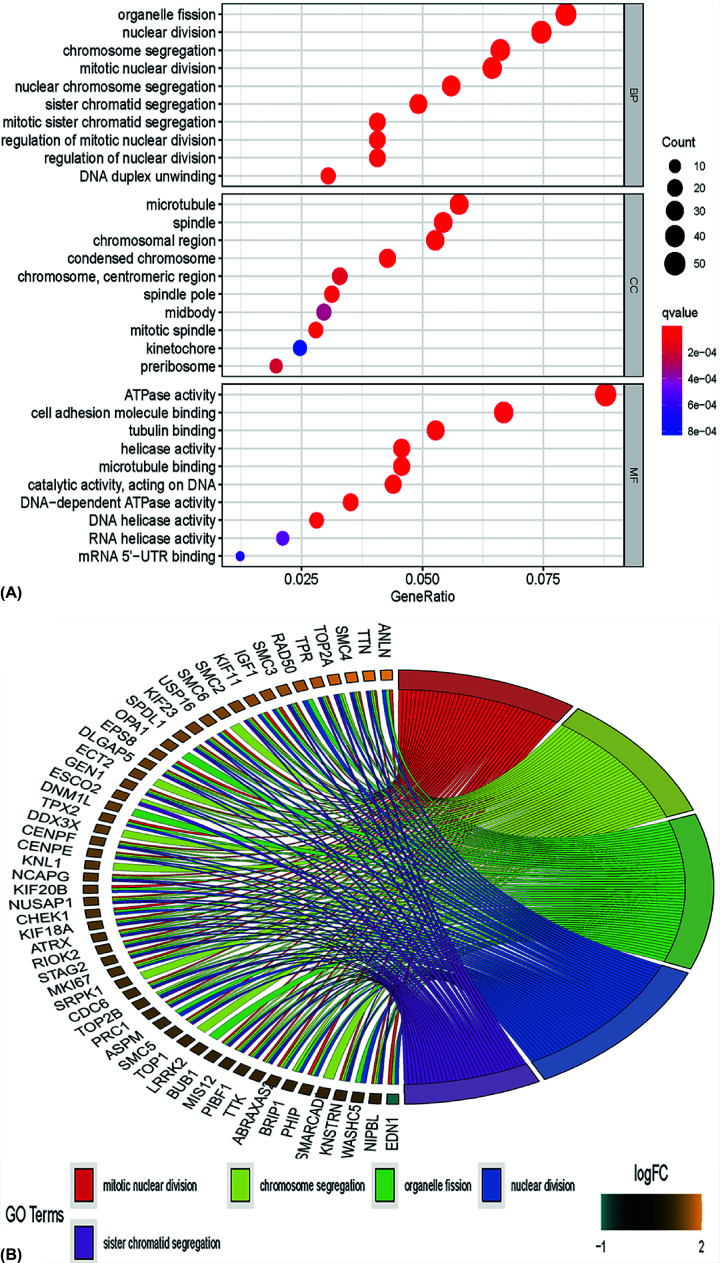
GO annotation results of DEGs (**A** and **B**) The horizontal axis represents the enriched gene ratio. The vertical axis represents the corresponding annotations on the most significantly enriched genes, including biological process, cellular component, and molecular function. The size of the circle indicates the number of enriched genes. The adjusted *P*-values are presented with the color scales by the side; DEG, differentially expressed gene; GO, Gene Ontology.

To determine biological features of DEGs, GO annotation was accomplished by R software (4.0.0) as follows: Most significant enrichment in Biological process (BP) of DEGs included mitotic nuclear division, organelle fission, chromosome segregation, and sister chromatid segregation. Primary enrichment in cell component (CC) of DEGs involved mitotic spindle, spindle, chromosomal region, and microtubule. Primary enrichment in molecule function (MF) consisted of ATPase activity, helicase activity, DNA-dependent ATPase activity, and DNA helicase activity (Figure 5A,B and Tables 1 and 2).

**Table 1:** GO and KEGG pathway enrichment analysis of DEGs in the PAH samples

Term	Description	Count	*P*-value
**Up-regulation**			
GO:0048285	Organelle fission	47	4.77E-15
GO:0000280	Mitotic nuclear division	44	5.72E-13
GO:0007059	Chromosome segregation	39	5.60E-13
GO:0005874	Microtubule	35	8.74E-08
GO:0005819	Spindle	33	1.10E-08
GO:0098687	Chromosomal region	32	3.82E-08
GO:0016887	ATPase activity	50	4.22E-15
GO:0050839	Cell adhesion molecule binding	38	8.53E-07
GO:0015631	Tubulin-binding	30	4.84E-07
hsa05168	Herpes simplex virus one infection	114	2.44E-07
hsa05412	Arrhythmogenic right ventricular cardiomyopathy	25	7.28E-05
hsa01212	Fatty acid metabolism	20	0.000113535
**Down-regulation**			
GO:0046777	Protein autophosphorylation	19	0.000180252
GO:0007051	Spindle organization	17	2.86E-05
GO:0090734	Site of DNA damage	8	0.001801353
GO:0097431	Mitotic spindle pole	5	0.001260684
GO:0005178	Integrin binding	14	9.05E-05
GO:0003725	Double-stranded RNA binding	9	0.000655078
hsa01212	Fatty acid metabolism	11	0.001127558
hsa01524	Platinum drug resistance	13	0.000232003

Abbreviations: DEG, differentially expressed gene; GO, Gene Ontology; KEGG, Kyoto Encyclopedia of Genes and Genomes; PAH, pulmonary arterial hypertension.

**Table 2:** GO and KEGG pathway enrichment analysis of DEGs in the most significant module

Term	Pathway description	Count	*P*-value
GO:0006996	Organelle organization	6	7.28E-05
GO:0000280	Nuclear division	5	5.88E-07
GO:0022402	Cell cycle process	5	5.62E-06
GO:0043229	Intracellular organelle	6	0.0219599139512363
GO:0043234	Protein complex	5	0.00510417758391666
GO:0005694	Chromosome	4	5.31E-04
GO:1901363	Heterocyclic compound binding	6	0.00719682984698825
GO:0032559	Adenyl ribonucleotide binding	5	5.57E-04
GO:0016887	ATPase activity	3	0.00687545528482454
hsa05168	Herpes simplex virus one infection	15	0.000113535
hsa01212	Fatty acid metabolism	19	0.000206574
hsa03013	RNA transport	10	0.000466728

Abbreviations: GO, Gene Ontology; DEG, differentially expressed gene; KEGG, Kyoto Encyclopedia of Genes and Genomes; PAH, pulmonary arterial hypertension.

**Table 3:** Features and functional roles of 10 hub genes screened from DEGs

No.	Gene symbol	Full name	Function
1	SMC4	Structural maintenance of chromosomes 4	SMC4 potentially promote response to innate inflammatory immune [23].
2	TOP2A	DNA topoisomerase II alpha	TOP2A decatenate intertwined DNA during anaphase to allow chromosome segregation to occur before cell division [33].
3	SMC2	Structural maintenance of chromosomes 2	SMC2 involving in chromosome segregation and stability chromosomal [20].
4	KIF11	Kinesin family member 11	Overexpression of KIF11during mitosis results in premature separation of sister chromatids and an uneven distribution of chromosomes [28].
5	KIF23	Kinesin family member 23	KIF23 plays an essential role in the bundling and transport of microtubules to specific intracellular locations in different cells at specific time points [34].
6	ANLN	Anillin actin-binding protein	ANLN is an F-actin binding protein that modulates podocyte cell motility and interacts with the phosphoinositide 3-kinase (PI3K) pathway via the slit diaphragm adaptor protein CD2-associated protein (CD2AP) [32].
7	ARHGAP11A	Rho GTPase activating protein 11A	ARHGAP11A dynamically regulated colon cancer cell motility and invasion and directly interacted with p53 tetramerization domain to exhibit a Rho-independent role in cancer [27].
8	SMC3	Structural maintenance of chromosomes 3	Smc3 acetylation stabilizes cohesin association with chromosomes, and its deacetylation by Hos1 in anaphase allows reuse of Smc3 in the next cell cycle [35].
9	SMC6	Structural maintenance of chromosomes 6	Smc5–Smc6 play an essential role in cellular processes such as genome replication, mitotic and meiotic chromosome segregation, DNA repair [30].
10	RAD50	RAD50 double-strand break repair protein	Homologs of Rad50 and Mre11 form Mre112-Rad50 hetero-tetramers, where two Rad50 ATP-binding cassette nucleotide-binding domains and a Mre11 nuclease dimer assemble as a catalytic head module that binds and cleaves DNA [22].

**Table 4:** Primer sequences for qRT-PCR

No.	Gene	Primers sequences (5′to3′)
1	Rat-SMC6-91F	TCTGATTGACATGCGGAGCA
2	Rat-SMC6-91R	TTTGGGTGGCTTTTGGGACT
3	Rattus-Top2a-197 F	CTGCCCATGCTCCCAAGTTA
4	Rattus-Top2a-197 R	GGTGTCTTCTCGGTGCCATT
5	Rattus-Kif11-182F	GGCAGCCAAAAGGACAACTG
6	Rattus-Kif11-182R	CAGCTCCAGAACGCCCAATA
7	Rattus-Kif23-160F	GGGGAAGGTTCGGATGATCG
8	Rattus-Kif23-160R	GTTTCTGTACCGTCTCCCCG
9	Rattus-Anln-155F	GACCCGTTCACCGAGAAGTT
10	Rattus-Anln-155 R	GGCTGCTGCTGATTACTTGC
11	Rattus-Arhgap11a-83F	TGCTAGACAACGGTCACTGG
12	Rattus-Arhgap11a-83R	CAGAAACCTTCCCGTGCTCT
13	Rattus-Smc3-131 F	AGGGACACTGCCTATCCTGA
14	Rattus-Smc3-131 R	GAAACCTCCATGCTCCGACA
15	Rat-α-tubulin-132 F	AGCGCCCAACCTACACTAACTTAAA
16	Rat-α-tubulin-132 R	GAGGGTAGGGCACCAGGTTG

Note: Tubulin regard as the reference gene.

### KEGG enrichment analysis

**Figure 6:**
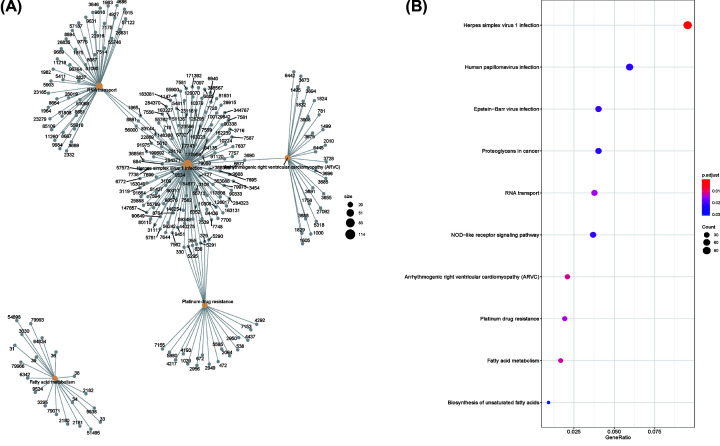
KEGG enrichment result of DEGs (**A** and** B**) The size of circle represents gene count. KEGG enrichment analysis of DEGs showed that many of these genes were mapped to Herpes simplex virus 1 infection, arrhythmogenic right ventricular cardiomyopathy (ARVC), fatty acid metabolism; DEG, differentially expressed gene; FDR, false discovery rate; KEGG, Kyoto Encyclopedia of Genes and Genomes.

To explore enriched pathways of DEGs, KEGG pathway analysis was done using R software (4.0.0). The result of investigation revealed that DEGs were mainly enriched in Herpes simplex virus one infection, Arrhythmogenic right ventricular cardiomyopathy (ARVC), fatty acid metabolism, RNA transport, and platinum drug resistance (Figure 6A,B).

### miRNA–gene inter-regulation network

**Figure 7:**
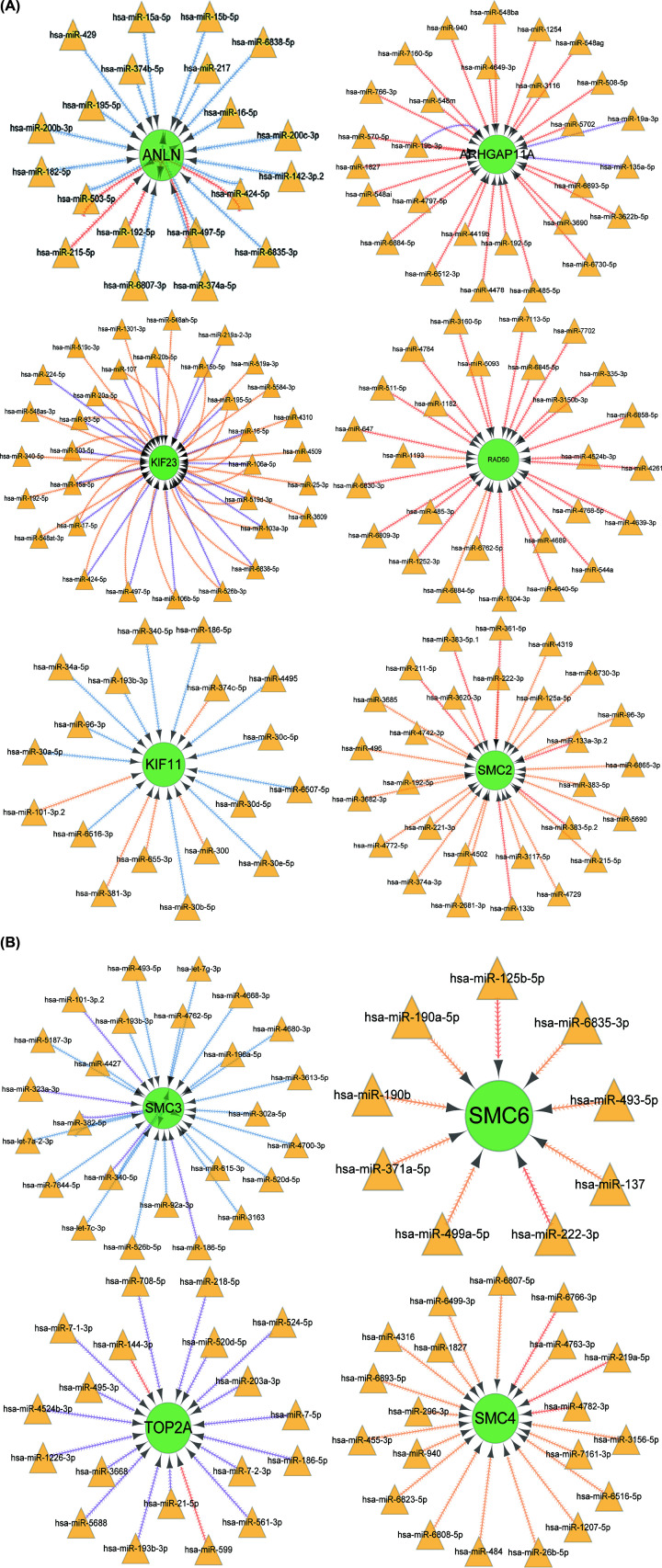
MiRNA–genes interaction network (**A** and** B**) Circles represent target genes with green color, and triangles symbolize miRNA with brown color.

For further explore the mechanism of the ten core genes, we investigated the potential interaction network of these genes and its response miRNA. An online platform, for instance, miRWalk, miRDB, and TargetScan were then to predict miRNA–mRNA interaction network and visualized through Cytoscape software (Figure 7).

### The analysis of qRT-PCR for hub genes

**Figure 8:**
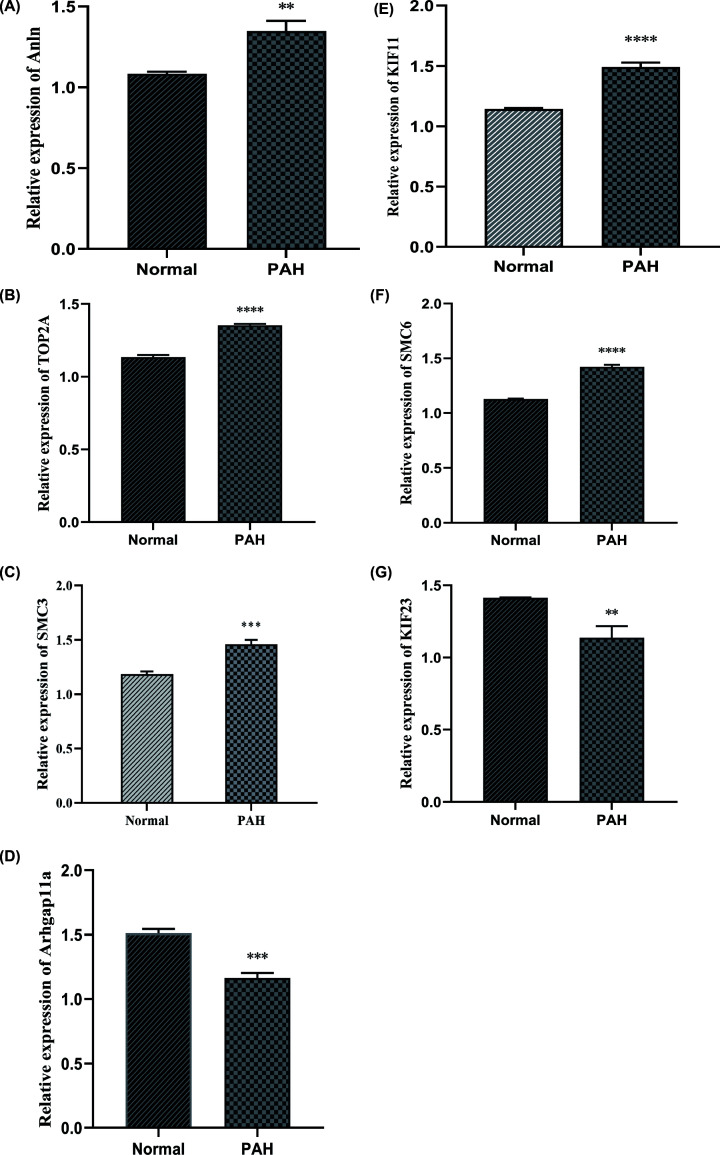
The results of qRT-PCR for top seven genes (**A–G**) The expression of SMC3, SMC6, Arhgap11A, KIF23, KIF11, TOP2A, and Anln determined by qRT-PCR; PAH, pulmonary arterial hypertension. **P<0.05, ***P<0.01, ****P<0.001.

In our study, ten hub genes were identified using the PPI network, which is of the essence to the pathogenesis of PAH. Among these genes, SMC2, SMC4, and RAD50 were well established by reports that they were overexpressed in PAH patients or animal models compared with healthy cases. In consequence, SMC3, SMC6, KIF11, KIF23, TOP2A, ARHGAP11A, and Anln were interested genes and were validated by qRT-PCR. As shown in Figure 8, the outcomes that KIF23 and ARHGAP11A were lower expressed in PAH rat lung artery tissues than the healthy groups while SMC3, SMC6, KIF11, TOP2A, and Anln were higher expression compared with the healthy group, which was consistence with our predict results (Figure 8).

### ConnectivityMap (CMap) analysis

To predict possible drugs or molecules that may mitigate PAH, CMap analysis was applied to find relevant molecular compounds that can reverse the expression of DEGs in cell lines. As shown in Table 5, obviously, Trioxysalen, Repaglinide, and Fluocinonide are the most significant three compounds.

**Table 5:** Drugs or compounds screened by CMap analysis (Mean < −0.4 and *P* < 0.01)

CMap names	Mean	Enrichment	*P*	Specificity	Percent non-null
Trioxysalen	−0.578	−0.879	0.00052	0	100
Repaglinide	−0.575	−0.849	0.00095	0	100
Fluocinonide	−0.415	−0.726	0.0033	0.0112	80
Camptothecin	−0.566	−0.852	0.00649	0.1818	100
Alfaxalone	−0.56	−0.851	0.00661	0	100
Thioguanosine	−0.567	−0.76	0.00682	0.0211	100
Procaine	−0.461	−0.687	0.00693	0.0508	100
Trazodone	−0.554	−0.84	0.00817	0.0562	100
GW-8510	−0.472	−0.746	0.00828	0.2672	100
Bephenium hydroxynaphthoate	−0.453	−0.675	0.00845	0	100
Reserpine	−0.606	−0.837	0.00863	0.0201	100
Mitoxantrone	−0.514	−0.834	0.00903	0.0638	100
Medrysone	−0.459	−0.62	0.00963	0.173	83

**CMap:** ConnectivityMap;

**DEGs:** Differentially Expressed Genes.

## Discussion

PAH is a chronic refractory disease characterized by arterial lesions in the small- to medium-sized distal pulmonary artery associated with arterial muscularization, concentric endocardial thickening, and formation of plexiform lesions, leading to right ventricular hypertrophy and failure [2,13]. Even though extensive efforts have been made in this field during recent years, the underlying pathological mechanism of PAH remains mostly unknown. The reason may be related to the complexity and variety of human genes, and traditional PAH animal models cannot solve this problem fundamentally. Due to the rapid progress of high-throughput microarray technology and bioinformatic methods, we could make better insight into the critical genes associated with PAH and a deeper understanding of its pathogenesis [14].

Here, we processed a total of 18837 genes. GSEA software could provide valuable information on large-scale genes with a relatively smaller fold change. Then, we found that the PAH group was most positively correlated with enriched gene sets like transcription elongation factor complex, inclusion body, and axon cytoplasm while compared with the control group. Kuanghueih Chen et al. indicated that transcription elongation factor complex might be involved in the pathogenesis of PAH [15]. Besides, we identified 150 DEGs between the PAH patients and normal control based on two mRNA microarray data sets. Protein–protein co-expression network showed closely linked genes among DEGs. GO annotation result of DEGs demonstrated that organelle fission, nuclear division, and chromosome segregation were significantly enriched, which is well consistent with current research findings [16]. KEGG enrichment analysis of DEGs showed that many of these genes were mapped to Herpes simplex virus one infection, arrhythmogenic right ventricular cardiomyopathy (ARVC) [2,17], and fatty acid metabolism [18], which suggested a critical role of immune and inflammatory responses in pulmonary arterial hypertension [19].

A total of ten DEGs were identification as hub genes as follows: SMC4, TOP2A, SMC2, KIF11, KIF23, ANLN, ARHGAP11A, SMC3, SMC6, and RAD50. Verónica Dávalos et al. suggested that high levels of structural maintenance of chromosomes 2 (SMC2) may be required to allow WNT-driven cell proliferation which contribute a lot to the development of PAH and that SMC2 down-regulate could lead to tumor cell apoptosis [20,21]. Li et al. showed the physiological of peroxisome proliferator-activated receptor γ(PPARγ) and DNA damage response (DDR) by using pulmonary arterial hypertension (PAH) as a model that impaired PPARγ signalling pathway related to endothelial cell dysfunction and disrupted PPARγ-UBR5 (MRE11-RAD50-NBS1) interaction, heightened ATM interactor (ATMIN) expression and DNA lesions. Therefore, PPARγ-DDR dysfunction may explain the genomic instability and loss of endothelial homeostasis in PAH [22]. According to a study conducted by Qinlan Wang, Smc4, a core subunit of condensin, to potentially promote an inflammatory innate immune response. They suggested that knockdown of Smc4 inhibited Toll-like receptor-mediated production of proinflammatory cytokines such as IL-6, TNF-α in macrophages [22,23]. HMGB1-TLR4 signaling axis has been shown to stimulate neutrophil NADPH oxidase (NOX2) in both neutrophils and lung microvascular endothelial cells, and NOX2 has played essential roles in the pathogenesis of PH. HMGB1 induces macrophages to secrete proinflammatory cytokines in a TLR4-dependent way [24]. These literature supported the importance of above-stated hub genes.

To date, there is no relevant publication on such hub genes as TOP2A, KIF11, KIF23, ANLN, ARHGAP11A, SMC3 and SMC6. Among them, KIF23 and ARHGAP11A were down-regulated in pulmonary arterial hypertension patients, which might have a protective role in PAH. KIF23 is a nuclear protein that localizes to the interzone of mitotic spindles and acts as a plus-end-directed motor enzyme to control the cellular shape and biological processes such as motility, mitosis, intracellular vesicle transport, organization, and positioning of membranous organelles [25,26]. ARHGAP11A localizes to the plasma membrane in early mitosis and the equatorial membrane in anaphase is known as a regulator of cell cycle-dependent motility and directly interact with p53 tetramerization domain to exhibit a Rho-independent role in cancer [27]. The up-regulation of remaining hub genes might exacerbate the PAH. KIF11 is an evolutionarily conserved microtubule motor protein that functions in centrosome and chromosome dynamics in mitosis, KIF11 silencing induced increases in nuclear areas, micronucleus formation, DNA content and chromosome numbers that may contribute to the pathogenesis of cancer [28,29]. The principal activity of the SMC5/6 complex is the maintenance of nuclear genome stability by resolving complex structures and possibly acting as an antagonist of the cohesin complex TheSMC5/6complex exercise many functions, such as the control of unidirectional rDNA replication, neutralizing toxic DNA intermediates during replication, preventing homologous recombination between nonhomologous sequences [30,31]. Anillin actin-binding protein (ANLN) encodes an actin-binding protein that plays a role in cell growth and migration and regulates actin cytoskeletal dynamics in podocytes [32]. All in all, these predicted genes were experiment supported by qRT-PCR.

In summary, our study aimed to identify key molecules involved in the pathophysiology of pulmonary hypertension. About 150 DEGs and ten hub genes were screened via multiple-microarray analysis, which may become potential targets clinical diagnosis and treatment of PAH in the near term. Our research embraces several merits. First, we applied GSEA to identify gene sets and GO enrichment (biological process, cell component, and molecular function) with a statistically significant between the PAH groups and normal control. As a result, transcription elongation factor complex, inclusion body, and axon cytoplasm were determined most positively linked with PAH. Second, we scrutinized 150 DEGs and finally screened ten essential genes from PPI network, the inter-regulation network between ten target genes, and response miRNA was then performed to explore the potential mechanism of their biological function thoroughly. More importantly, these predicted molecules were well consolidated by experiment. However, owing to the lack of PAH patients’ detailed information, it is difficult to draw a clear association between selected genes and the severity of PAH while using the same samples. The mechanisms of PAH are needed to further explore *in vitro* or *in vivo* experiments.

## Data Availability

All datasets for this study are included in the article, and Supplementary Materials are available from the corresponding author.

## Abbreviations

CMap: ConnectivityMap
DEG: differentially expression gene
FDR: false discovery rate
GEO: gene expression omnibus
GO: gene ontology
GSEA: Gene set enrichment analysis
KEGG: Kyoto Encyclopedia of Genes and Genomes
PPI: protein–protein interaction
